# Childhood Allergy and Neurodivergence: A Cross‐Sectional Analysis in a UK‐Birth Cohort

**DOI:** 10.1111/all.70000

**Published:** 2025-08-13

**Authors:** Isra Husain, Somnath Mukhopadhyay, Jessica Eccles

**Affiliations:** ^1^ Department of Clinical and Experimental Medicine Brighton and Sussex Medical School Brighton UK; ^2^ Royal Alexandra Children's Hospital University Hospitals Sussex NHS Foundation Trust Brighton UK; ^3^ Department of Clinical Neuroscience Brighton and Sussex Medical School Brighton UK; ^4^ Sussex Partnership NHS Foundation Trust Worthing UK

**Keywords:** food allergy, paediatrics, personlized medicine

To the Editor,

1

Allergy emerges in childhood and has multisystemic and life‐threatening consequences [1]. Childhood allergy may be related to other conditions, and associations have been found between allergy and neurodivergence, including autism and/or attention‐deficit hyperactivity disorder (ADHD), with proposed mediatory mechanisms including immune dysfunction [2]. The association between allergy and autism has been demonstrated in Asia and the USA, but, to our knowledge, not in Europe (Supporting Information S1). Correspondingly, its association with ADHD has been shown in Asia, Europe and the United States but not in the United Kingdom (Supporting Information S1). Our aims were therefore to investigate the association between allergy and neurodivergence in a UK birth cohort.

Data were analysed from the Avon Longitudinal Study of Parents and Children cohort. In total, 15,450 study participants were enrolled at birth, with full Methods detailed in Supporting Information S2 [3, 4]. Variables of interest included the presence of likely autism at 0–3 years, determined at age 7 utilising the Social Communication Disorder Checklist [5], and likely ADHD at 9 years old, determined by the Strength and Difficulties Questionnaire hyperactivity scale [6]. Allergy‐related outcomes comprised the presence of food allergy from 7 years old and ‘other allergy’ (to non‐food substances) at 8 years old. Logistic regression analyses, adjusted for participant sex, were performed to investigate the association between allergy‐related outcomes and neurodivergence outcomes.

The numbers of participants are depicted in the Participants Flowchart (Supporting Information S3). Children with likely autism at 0–3 years had significantly increased odds of having a food allergy, compared to children unlikely to have autism (OR = 1.59 [95% CI = 1.15–2.19]; *p* = 0.005). Children with likely ADHD at 9 years old had significantly increased odds of having a food allergy, compared to children unlikely to have ADHD (OR = 1.82 [95% CI = 1.23–2.69]; *p* = 0.03).

However, children with likely autism at 0–3 years did not have significantly increased likelihood of having ‘other allergy’, compared to children unlikely to have autism (OR = 1.00 [95% CI = 0.81–1.24]; *p* = 0.999). Similarly, children with likely ADHD at 9 years old did not have significantly increased likelihood of having ‘other allergy’, compared to children unlikely to have ADHD (OR = 1.15 [95% CI = 0.89–1.50]; *p* = 0.28).

These results demonstrate significant associations between food allergy and likely autism and ADHD in a UK birth cohort. This emerging link between neurodivergence and allergy will be of interest to general practitioners and paediatricians, who are important points of healthcare contact for these patients and seek to provide holistic care.

The aetiologies of autism and ADHD are commonly accepted as multifactorial, with specific causative mechanisms poorly understood. Current evidence suggests elevated immune dysregulation, extending to neuroinflammation, in children with autism and ADHD compared to neurotypical children. As increased systemic inflammation is a hallmark of atopy, this may be a mediatory hypothesis (Supporting Information S4).

Regarding limitations, food allergy data was parent‐reported, which may have included reactions with non‐allergic aetiologies, such as intolerances and aversion. This may have contributed to our findings of association between food allergy and likely neurodivergence. Data also concerned reactions from 7 years old, which may have excluded allergies noted beforehand. Moreover, although variables of interest were collected from validated questionnaires, these indicated likelihood of autism or ADHD, rather than diagnoses.

In conclusion, our results support the evidence‐base indicating the need for integration of mind and body in neurodivergent children and young people. Future studies may wish to investigate the temporality of the relationship between allergy and neurodivergence and identify potential mediators. This could provide greater clarity regarding the characteristics of patient groups. This, in turn, could facilitate early identification of neurodivergence and design of personalised care pathways, helping to reduce the health inequalities faced by neurodivergent children and young people.

## Conflicts of Interest

The authors declare no conflicts of interest.

## Supporting information


Supporting Information S1.

Supporting Information S2.

Supporting Information S3.

Supporting Information S4.

## Data Availability

No new data was created from this research project. The data used in this paper is from the Avon Longitudinal Study of Parents and Children Repository as indicated in the reference section. Access to this data is through application only to the ALSPAC at alspac-data@bristol.ac.uk. [Correction added on 16 September 2025, after first online publication: Data Availability Statement has been updated]
