# Supplementary material for: Childhood Allergy and Neurodivergence: A Cross‐Sectional Analysis in a UK‐Birth Cohort
Source: Allergy. 2025 Aug 13;80(12):3452–3. doi: 10.1111/all.70000 (PMC12666756; doi:10.1111/all.70000)
Supplement: Supplementary file 1 — Supporting Information S1. [file ALL-80-3452-s002.docx]

**Existing Evidence [Supporting Information S1]**

Evidence from cross-sectional studies in Israel and the U.S.A report significant associations between one or more allergic diseases and autism_[1-4]_. Similar associations have been found between allergic disease and ADHD in Israel, Korea, Taiwan, and the U.S.A_[4-7]_. This is supported by findings from prospective studies in Taiwan, which suggest significant associations between asthma, allergic rhinitis and atopic dermatitis and development of autism_[8-11]_. When investigating for association between the childhood atopy and development of ADHD, this is supported by cohort studies conducted in Germany, Sweden, and Taiwan_[8-10][12-13]_. One cross-sectional study on children in Turkey, however, did not find correlation between clinical signs of atopy and autism_[14]_. These results may have been influenced by the small sample size of this study.

**References**

1. Xu G, Snetselaar LG, Jing J, Liu B, Strathearn L, Bao W. Association of Food Allergy and Other Allergic Conditions with Autism Spectrum Disorder in Children. JAMA Network Open. 2018;1(2):e180279.
2. Yadama AP, Kely RS, Lee-Sarwar K, Mirzakhani H, Chu SH, Kachroo P, Litonjua AA et al. Allergic disease and low ASQ communication score in children. Brain, Behavior, and Immunity. 2020;83:293-7.
3. Kotey S, Ertel K, Whitcomb, B. Co-occurrence of Autism and Asthma in a Nationally-Representative Sample of Children in the United States. Journal of Autism and Developmental Disorders. 2014;44(12):3083-8.
4. Nemet S, Asher I, Yoles I, Baevsky T, Sthoeger Z. Early childhood allergy linked with development of attention deficit hyperactivity disorder and autism spectrum disorder. Pediatric Allergy and Immunology. 2022;33(6).
5. Tsai JD, Chang SN, Mou CH, Sung FC, Lue KH. Association between atopic diseases and attention-deficit/hyperactivity disorder in childhood: A population-based case-control study. Annals of Epidemiology. 2013;23(4):185-8.
6. Strom MA, Fishbein AB, Paller AS, Silverberg JI. Association between atopic dermatitis and attention deficit hyperactivity disorder in U.S. children and adults. British Journal of Dermatology, 2016;175(5):920-9.
7. Kwon HJ, Lee MY, Ha M, Yoo SJ, Paik KC, Lim JH, Sakong J et al. (2014) “The associations between ADHD and asthma in Korean children,” BMC Psychiatry. 2014;14(1).
8. Li DJ, Tsai CS. Hsiao RC, Chen YL, Yen CF. Associations between Allergic and Autoimmune Diseases with Autism Spectrum Disorder and Attention-Deficit/Hyperactivity Disorder within Families: A Population-Based Cohort Study. International Journal of Environmental Research and Public Health. 2022;19(8):4503.
9. Lee CY, Chen MH, Jeng MJ et al. Longitudinal association between early atopic dermatitis and subsequent attention-deficit or autistic disorder A population-based case-control study. Medicine (Baltimore). 2016;95(39):e5005
10. Liao TC, Lien YT, Wang S, Huang SL, Chen CY. Comorbidity of Atopic Disorders with Autism Spectrum Disorder and Attention Deficit/Hyperactivity Disorder. Journal of Pediatrics. 2016;171:248-55.
11. Tsai PH, Chen MH, Su TP et al. Increased risk of autism spectrum disorder among early life asthma patients: An 8-year nationwide population-based prospective study. Research in Autism Spectrum Disorders. 2014;8(4):381-6.
12. Genuneit J, Braig S, Brandt S et al. Infant atopic eczema and subsequent attention-deficit/hyperactivity disorder - A prospective birth cohort study. Pediatric Allergy and Immunology. 2014;25(1):51-6.
13. Lichtenstein P, Tuvblad C, Larsson H, Carlstrom E. The Swedish Twin study of CHild and Adolescent Development: The TCHAD-study. Twin Research and Human Genetics. 2007;10(1):67-73.
14. Bakkaloglu B, Anlar B, Anlar FY et al. Atopic features in early childhood autism. European Journal of Paediatric Neurology. 2008;12(6):476-9.
