# Supplementary material for: Childhood Allergy and Neurodivergence: A Cross‐Sectional Analysis in a UK‐Birth Cohort
Source: Allergy. 2025 Aug 13;80(12):3452–3. doi: 10.1111/all.70000 (PMC12666756; doi:10.1111/all.70000)
Supplement: Supplementary file 2 — Supporting Information S2. [file ALL-80-3452-s004.docx]

**Methods [Supporting Information S2]**

**Participants**

Data was utilized from the Avon Longitudinal Study of Parents and Children (ALSPAC), a prospective birth cohort study_._ The core sample, termed ‘G1’, was a cohort of the children of pregnant women with expected delivery dates between April 1991 and December 1992 in Avon, south-west England.

Pregnant women resident in Avon, UK with expected dates of delivery between 1st April 1991 and 31st December 1992 were invited to take part in the study. The initial number of pregnancies enrolled was 14,541, resulting in 14,676 foetuses, 14,062 live births and 13,988 children alive at 1-year of age. When the oldest children were approximately 7 years of age, eligible cases who had failed to join the study originally were recruited. The total sample size for analyses using any data collected after the age of seven is 15,447 pregnancies, resulting in 15,658 foetuses. Of these, 14,901 children were alive at 1 year of age. Children were followed up at regular intervals and data was collected by parent and/or child-completed questionnaires and biological samples. At age 18, study children were sent 'fair processing' materials describing ALSPAC’s intended use of their health and administrative records and were given clear means to consent or object via a written form. Data were not extracted for participants who objected, or who were not sent fair processing materials. The study website contains details of all the data that is available through a fully searchable data dictionary and variable search tool_[13]_.

**Ethical approval**

Ethical approval for the study was obtained from the ALSPAC Ethics and Law Committee and the Local Research Ethics Committees (NHS Haydock REC: 10/H1010/70). Informed consent for the use of data collected via questionnaires and clinics was obtained from participants following the recommendations of the ALPSAC Ethics and Law Committee at the time.

**Variables**

Variables described below were derived from questionnaires completed by mothers of children at different timepoints.

***Presence of food allergy from age 7 years***

Presence of food allergy from age seven was identified through the ‘My Son/Daughter’s Health’ questionnaire, which was completed by mothers when their children were 8-years old. This questionnaire collected information regarding children’s physical wellbeing.

For the purposes of our analyses, we utillised data from section C (Allergies) to ascertain parent-reported allergic reaction to food and/or drink since their child’s seventh birthday. We re-coded these answers into binary variables ‘yes possibly or definitely’ or ‘definitely none’.

***Presence of other allergy***

Presence of allergy to substances other than food and drink was also identified from Section C (Allergies) from the ‘My Son/Daughter’s Health’ questionnaire_[13]._ The question asked what the child was currently allergic to, other than food or drink. We re-coded answers into binary variables ‘other allergy’ or ‘no other allergy’.

***Presence of any allergy***

Presence of ‘any allergy’ was defined as presence of food allergy from 7 years and/or presence of allergy to substances other than food and drink, as described above. We re-coded answers into binary variables ‘any allergy’ or ‘no allergy’.

**Main outcome measures**

***Neurodivergence***

The Social Communication Disorder Checklist (SCDC)_[13]_ was completed by mothers when children were 7-years old, concerning their children’s behaviour at ages 0-3. The SCDC is a validated questionnaire which assesses communication skills and social reciprocity. It is a 12-item scale, scored between 0-24, with higher scores indicating increased likelihood of autistic traits_[14]._ We calculated variables for presence of likely autism, using the widely recommended cut-off score of ≥ 9_[15]_.

The Strengths and Difficulties Questionnaire (SDQ)_[16]_, a validated questionnaire comprising five subscales of ‘difficulties’ faced by children, was completed by mothers when children were 9-years old. We used the hyperactivity subscale, scored between 0-40, with a higher score suggesting a greater degree of hyperactivity-related difficulties. We calculated variables for presence of likely ADHD using the recommended cut-off score of ≥ 8 on SDQ hyperactivity scale_[17]_.

For SCDC and SDQ questionnaires, scores were prorated to account for any missing data.

We calculated a composite outcome measure, termed ‘neurodivergence score’, for each participant, indicating presence of likely autism and/or likely ADHD in children. This was derived from the average of Z-scores from each participant’s SCDC and SDQ hyperactivity scale.

**Statistical analyses**

Statistical analysis was conducted using SPSS Statistics Version 29.0.2.0. We used logistic regression to investigate the relationship between allergy and likely autism, and allergy and likely ADHD (as separate analyses) by calculating odds ratios (ORs) and 95% confidence intervals (CIs). Linear regression was used to investigate whether the presence of allergy in childhood predicted neurodivergence score.
