# Supplementary material for: Childhood Allergy and Neurodivergence: A Cross‐Sectional Analysis in a UK‐Birth Cohort
Source: Allergy. 2025 Aug 13;80(12):3452–3. doi: 10.1111/all.70000 (PMC12666756; doi:10.1111/all.70000)
Supplement: Supplementary file 3 — Supporting Information S3. [file ALL-80-3452-s001.docx]

**Supporting Information S3: Participants Flowchart**, illustrating the stages of data collection and number of participants (n) at each stage

*SCDC = social communication disorders checklist; SDQ = strengths and difficulties questionnaire*

SDQ

Complete data (n = 8048)

SCDC

Complete data (n = 8100)

Not likely ADHD

95.4% (n = 7675)

Likely ADHD

4.6% (n = 373)

**Your Son/Daughter At 9** at **Age 9**

Questionnaires returned (n = 8227)

Food allergy not present 93.7% (n = 7391 )

Other allergy not present 77.5% (n =6276 )

Other allergy present 22.5%(n = 1826 )

22.5

Food allergy present 6.3% (n = 496 )

Other allergy

Complete data (n = 7348 )

Food allergy

Complete data (n = 7887 )

Not likely autism

48.4% (n = 7471)

Likely autism

7.8% (n = 629 )

**My Son/Daughter’s Health**

**(0-7years)** at **Age 8**

Questionnaires returned (n = 8339)

**ALSPAC sample alive at age 1**

Core sample + non-core sample

(n = 14,901)

**My Son/Daughter’s Wellbeing**

at **Age 7**

Participants assessed (n = 8269)
