# Supplementary material for: Childhood Allergy and Neurodivergence: A Cross‐Sectional Analysis in a UK‐Birth Cohort
Source: Allergy. 2025 Aug 13;80(12):3452–3. doi: 10.1111/all.70000 (PMC12666756; doi:10.1111/all.70000)
Supplement: Supplementary file 4 — Supporting Information S4. [file ALL-80-3452-s003.docx]

**Supporting Information S4 – Mechanisms of Association (Discussion)**

Regarding the aetiologies of autism and ADHD, there is limited evidence, with a multifactorial model likely. Growing evidence supports presence of immune dysregulation and neuroinflammation in both conditions compared to neurotypical individuals, with evidence of increased levels of interleukins -1β, -6, and -8, tumour necrosis factor, and monocyte chemoattractant protein-1 in the brain and cerebrospinal fluid of autistic individuals_[1][2]_. In individuals with ADHD, studies report elevated levels of interleukin-10 and central nervous system autoantibodies _[3-6]_. As increased systemic inflammation is a hallmark of atopic conditions_[7]_, this may be a possible hypothesis linking atopy and neurodivergence.

**References**

1. Ashwood P, Krakowiak P, Hertz-Picciotto, I, Hansen, R, Pessah, I, van de Water, J. Elevated plasma cytokines in autism spectrum disorders provide evidence of immune dysfunction and are associated with impaired behavioral outcome. Brain, Behavior, and Immunity. 2011;25(1):40-45.
2. Li X, Chauhan, A, Sheikh AM, Patil S, Chauhan V, Li XM, Ji L et al. Elevated immune response in the brain of autistic patients. Journal of Neuroimmunology. 2009;207(1–2):111-116.
3. Darwish AH., Elgohary TM, Nosair NA. Serum Interleukin-6 Level in Children With Attention-Deficit Hyperactivity Disorder (ADHD). Journal of Child Neurology. 2019;34(2):61-67. Available at: https://doi.org/10.1177/0883073818809831
4. Donfrancesco R, Nativio P, di Benedetto A, Villa MP, Andriola E, Melegari MG, Cipriano, E et al. Anti-Yo Antibodies in Children With ADHD: First Results About Serum Cytokines. Journal of Attention Disorders. 2020;24(11): 1497-1502. Available at: https://doi.org/10.1177/1087054716643387
5. Martins-Silva T, Vaz JDS, Hutz MH, Salatino-Oliveira, A, Genro JP, Hartwig FP, Moreira-Maia, C L et al. Assessing causality in the association between attention-deficit/hyperactivity disorder and obesity: a Mendelian randomization study. International Journal of Obesity. 2019;43(12):2500-2508. Available at: https://doi.org/10.1038/s41366-019-0346-8
6. Welsh P, Polisecki, E., Robertson M, Jahn S, Buckley BM, de Craen, A.JM, Ford, I., et al. Unraveling the directional link between adiposity and inflammation: A bidirectional mendelian randomization approach. Journal of Clinical Endocrinology and Metabolism. 2010;95(1):93-99. Available at: <https://doi.org/10.1210/jc.2009-1064>
7. Zheng T, Yu J, Oh MH, Zhu Z. The Atopic March: Progression from Atopic Dermatitis to Allergic Rhinitis and Asthma. Allergy Asthma Immunol Res. 2011;3(2):67-73. Available at: <https://doi.org/10.4168/aair.2011.3.2.67>
